# Psychosocial predictors for outcome after total joint arthroplasty: a prospective comparison of hip and knee arthroplasty

**DOI:** 10.1186/s12891-018-2058-y

**Published:** 2018-05-22

**Authors:** Marion Lindner, Olaf Nosseir, Anett Keller-Pliessnig, Per Teigelack, Martin Teufel, Sefik Tagay

**Affiliations:** 1 Department of Psychosomatic Medicine and Psychotherapy, University of Duisburg-Essen, Virchowstraße 174, 45147 Essen, Germany; 2 Department of General, Orthopedic and Accident Surgery, Sankt Josef-Hospital GmbH, In der Hees 4, 46509 Xanten, Germany

**Keywords:** Total hip arthroplasty, Total knee arthroplasty, Health-related quality of life, Psychological distress, Psychosocial resources

## Abstract

**Background:**

As findings regarding predictors for good outcome after total joint arthroplasty are highly inconsistent, aim of this study was to investigate the influence of the psychosocial variables sense of coherence and social support as well as mental distress on physical outcome after surgery. It should be investigated if different predictors are important in patients after total hip arthroplasty (THA) compared to patients after total knee arthroplasty (TKA).

**Methods:**

In a prospective design, 44 patients undergoing THA and 61 patients undergoing TKA were examined presurgery and 6 and 12 weeks after surgery using WOMAC (disease-specific outcome), SF-36 (health-related quality of life), BSI (psychological distress), SOC-13 (sense of coherence), and F-SozU (social support). Changes over time were calculated by analyses of variance with repeated measures. Stepwise multiple linear regression analyses were computed for each group to predict scores of WOMAC total and all WOMAC subscales 12 weeks postoperatively.

**Results:**

THA as well as TKA patients experienced improvements in all parameters (effect sizes for WOMAC scores between η^2^ = .387 and η^2^ = .631) with THA patients showing even better results than TKA patients. WOMAC scores 12 weeks after surgery were predicted predominantly by WOMAC baseline scores in TKA with an amount of explained variance between 9.6 and 19.5%. In THA, 12-weeks WOMAC scores were predicted by baseline measures of psychosocial aspects (anxiety, sense of coherence, social support). In this group, predictors accounted for 17.1 to 31.6% of the variance.

**Conclusions:**

Different predictors for outcome after total joint arthroplasty were obtained for THA and TKA patients. Although psychosocial aspects seemed to be less important in TKA patients, preoperatively, distressed patients of both groups should be offered interventions to reduce psychological distress to obtain better outcomes after surgery.

## Background

Besides objective medical parameters like mortality, prosthesis survival or range of motion, health-related quality of life (HRQoL) is an important outcome of surgical interventions. A large number of studies investigated HRQoL after total knee (TKA) or total hip (THA) arthroplasty and reported impressing results with highly significant increases in general HRQoL as well as in disease-specific outcome [1–6]. Nevertheless, a small proportion of patients do only reach poor or no improvement, raising the question which determinants could have an impact on joint replacement outcome. For this reason, sociodemographic and psychosocial variables have been taken into account, investigating their potential contribution to HRQoL after TKA or THA. For example, educational status, social support, depression [7] and mental distress [2] have been found to influence follow-up pain and physical function scores. As another widely used psychological parameter, sense of coherence (SOC) was shown to play an important role in perceived health and HRQoL of individuals with and without physical or mental illness, independent of age, sex, nationality and ethnicity [8, 9]. SOC, proclaimed by Antonovsky [10], describes “a global orientation that expresses the extent to which one has a pervasive, enduring though dynamic feeling of confidence” (p. 19), and consists of the three components comprehensibility, manageability and meaningfulness. In patients with osteoarthritis, a high baseline SOC was associated with a better mental as well as physical HRQoL after THA [11], yet SOC is generally stronger related to mental aspects of health [8, 9, 12].

Results of studies which evaluated THA and TKA patients separately suggest that patients´ benefit after THA is even greater with respect to pain, HRQoL, function and satisfaction than after TKA [3, 6, 13–15], but there are also different findings [16]. A review investigating psychological factors affecting the outcome of total joint arthroplasty [17] summarized that evidence is strong regarding TKA whereas results for THA are limited and conflicting.

Against this background, the purpose of this study is to examine the influence of the psychosocial resources SOC and social support as well as of mental distress on outcome separately in patients after THA and TKA to define different predictors in these two patient groups.

## Methods

### Study design

Over a period of 1 year, patients with either osteoarthritis of the hip or with osteoarthritis of the knee were consecutively recruited in the surgical ward of a German hospital. Patients had been diagnosed by clinical and radiological examinations, and total replacement of hip or knee was indicated. All knee arthroplasties were performed with cemented technique, hip arthroplasties were performed with uncemented technique whenever possible due to bone quality. Surgeries were predominantly performed by the second author. At the day of admission to the hospital, patients were asked for study participation. Inclusion criteria were indication of total joint arthroplasty due to either coxarthrosis or gonarthrosis, sufficient knowledge of German language to complete the questionnaires and voluntary study participation. Exclusion criteria were osteoarthritis of both hip and knee, contralateral arthroplasty or another joint arthroplasty, any neurological diseases like muscle weakness, Parkinson’s disease or (suspected) dementia, movement restrictions caused by any other reason than osteoarthritis, joint infections, fractures and prosthesis replacements. After confirming written informed consent, patients filled in a questionnaire collecting sociodemographic data as well as the self-rating instruments WOMAC (disease-specific outcome), SF-36 (health-related quality of life), BSI (psychological distress), SOC-13 (sense of coherence), and F-SozU (social support) the first time (t0). Hospital length of stay was 12 days. Subsequently, all patients were referred to a rehabilitation clinic for treatment between 3 and 4 weeks. Six weeks (t1) as well as 12 weeks (t2) after surgery, patients returned to hospital for medical check-up and completed the same questionnaires again. The study was approved by the hospital’s ethics committee.

### Instruments

#### Western Ontario and Mac Master Universities Osteoarthritis Index (WOMAC) [18, 19]

The WOMAC is an internationally widely used reliable and valid instrument that measures disease-specific dimensions in patients with osteoarthritis in lower limb. It consists of 24 items which are grouped into the subscales pain, stiffness and functional limitations (function), altogether building the total score. The items are answered on an 11-point Likert-scale. A high value suggests a high degree of symptoms. At t0, the subscale pain showed an internal consistency of α = .88, stiffness of α = .82, function of α = .95 and the total score of α = .96.

#### Short Form-36 Health Survey (SF-36) [20, 21]

SF-36 is a psychometrically proven questionnaire for the assessment of general HRQoL. The 36 items are divided into eight subscales and a physical (PCS) as well as a mental (MCS) component summary score can be computed. A high value suggests a high HRQoL. In this study, the internal consistency of the subscales ranged from α = .61 to α = .93 at t0.

#### Brief Symptom Inventory (BSI) [22]

The BSI, a short form of the SCL-90-R, measures the subjective disturbance by physical and mental symptoms. The 53 items are summed up to nine subscales and three global values. In this study, the subscales anxiety and depression as well as the global severity index (GSI) were analyzed. High values suggest high psychological distress. At t0, the internal consistency for depression was α = .88, for anxiety α = .78 and for the whole questionnaire α = .96.

#### Sense of coherence scale-13 (SOC-13) [23]

The SOC-13, a short form of the SOC-29, measures the extent of the sense of coherence as proclaimed by Antonovsky [10]. The 13 items are answered on a 7-point Likert-scale. A high value suggests a high sense of coherence. At t0, the SOC-13 showed an internal consistency of α = .74.

#### Questionnaire for social support (F-SozU) [24]

F-SozU is used for the assessment of subjectively perceived social support. The short form consists of 22 items, which are answered on a 5-point Likert-scale. A high value suggests high social support. The internal consistency of this questionnaire was α = .92 at t0.

### Statistical analysis

Data were analyzed using the software SPSS 22.0 for Microsoft Windows®. For descriptive analyses, data were expressed as mean values (M) and standard deviation (SD). Mean differences of variables between THA and TKA patients were investigated by t-tests for independent samples and Chi-square-tests. Scores of all instruments except of WOMAC were compared with healthy reference groups of the questionnaire’s manuals or from the literature by use of one sample t-tests (SF-36: *n* = 2773, healthy adults; BSI: *n* = 600, healthy adults; SOC-13: *n* = 1944, representative German sample; F-SozU: *n* = 2179, representative German sample). Analyses of variance with repeated measures were calculated to find changes over time, group effects (THA vs. TKA) and interaction effects between time and group. As measure for the effect size, eta-squared (η^2^) was used. Values of eta-squared are interpreted as follows: η^2^ = 0.01: small, η^2^ = 0.09: medium, η^2^ = 0.25: large. With stepwise multiple linear regression analyses the relative contribution of possible predictors to the outcome parameters WOMAC pain, WOMAC stiffness, WOMAC function and WOMAC total have been investigated for each group separately. As independent variables the baseline scores of BSI anxiety, BSI depression, SOC-13, F-SozU and of each model’s dependent variable have been included. As there was no significant correlation between age and the outcome parameters and because no differences were found between males and females regarding the WOMAC scales, age and gender were not included as independent variables in the regression models. For comparisons with reference samples, α-level was corrected for multiple testing (2 groups × 3 time points: α = .05/6 = .008). For all other tests, a significance level of α ≤ .05 was predetermined.

## Results

### Sample

Overall, 119 consecutive TKA and THA patients were screened for eligibility and asked for participation in the study. Seven patients (5.9%) declined participation and two patients (1.7%) did not meet inclusion criteria, thus 110 patients (92.4%) were included. During the study period, two patients rejected participation (1.8%), one patient (0.9%) could not continue because of health reasons independently of joint arthroplasty and two patients (1.8%) lacked to complete the questionnaires so that 105 data sets were analyzed.

Of the patients, 41.9% had osteoarthritis of the hip, in the other 58.1%, the knee was affected. Mean age was 67.4 years (SD = 10.3) for THA and 66.4 years (SD = 10.6) for TKA patients. In the THA sample, 52.3% were female, in the TKA sample 60.7%. Both groups only differed significantly from each other regarding the marital status (χ ^2^ = 8.499; *p* = .037). Table 1 shows the sociodemographic data separately for THA and for TKA patients.

**Table 1:** Sociodemographic data of THA and TKA patients

	THA: *n* = 44 (41.9%)	TKA: *n* = 61 (58.1%)	
Age	M = 67.4 (SD = 10.3)	M = 66.4 (SD = 10.6)	*t* = 0.479; *p* = .633
Sex			Χ^2^ = 0.734; *p* = .392
Male	21 (47.7%)	24 (39.3%)	
Female	23 (52.3%)	37 (60.7%)	
Marital status			Χ^2^ = 8.499; *p* = .037
Single	1 (2.3%)	8 (13.1%)	
Married	39 (88.6%)	40 (65.6%)	
Separated/divorced	0	3 (4.9%)	
Widowed	4 (9.1%)	10 (16.4%)	
Living situation			Χ^2^ = 5.459; *p* = .243
Alone	3 (6.8%)	10 (16.4%)	
With partner	29 (65.9%)	37 (60.7%)	
With children	1 (2.3%)	4 (6.6%)	
With partner and children	10 (22.7%)	7 (11.5%)	
Others	1 (2.3%)	3 (4.9%)	
Partnership			Χ^2^ = 3.032; *p* = .082
Yes	39 (88.6%)	45 (73.8%)	
No	4 (9.1%)	13 (21.3%)	
Unknown	1 (2.3%)	3 (4.9%)	
Education			Χ^2^ = 5.406; *p* = .248
No certificate	2 (4.5%)	6 (9.8%)	
Certificate of secondary education	26 (59.1%)	40 (65.6%)	
General certificate of secondary education	9 (20.5%)	12 (19.7%)	
University certification	1 (2.3%)	1 (1.6%)	
College degree	5 (11.4%)	1 (1.6%)	
Unknown	1 (2.3%)	1 (1.6%)	
Employment status			Χ^2^ = 4.002; *p* = .549
Employed	10 (22.7%)	17 (27.8%)	
Housewife/−husband	11 (25.0%)	15 (24.6%)	
Retired	23 (52.3%)	27 (44.3%)	
Unknown	0	2 (3.3%)	

Legend: *THA* total hip arthroplasty, *TKA* total knee arthroplasty, *M* mean value, *SD* standard deviation

With respect to psychometric instruments, patient groups showed no significant differences in all WOMAC scales, SF-36 PCS, BSI anxiety and depression, SOC-13, and F-SozU before surgery. However, THA patients showed higher SF-36 MCS (*t* = 2.43; *p* = .017) and lower BSI GSI scores (*t* = − 2.03; *p* = .046) than TKA patients.

### Changes in disease-specific dimensions

**Fig. 1:**
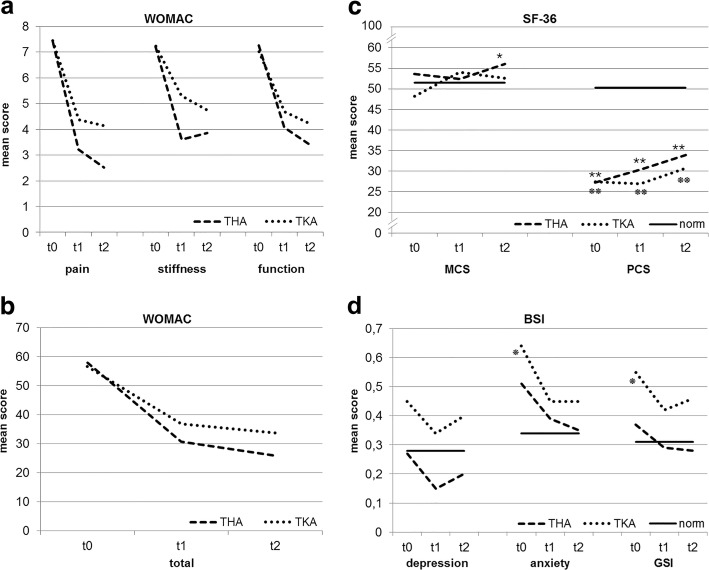
Changes in THA and TKA patients over 12 weeks and comparisons with healthy reference groups. **a** THA and TKA patients show improvements on all WOMAC subscales. **b** THA and TKA patients show improvements on the WOMAC total score. **c** Mental HRQoL (MCS) showed an overall improvement in TKA but not in THA patients. Physical HRQoL (PCS) improved in both groups. **d** THA and TKA patients improved on all three BSI scales. BSI=Brief Symptom Inventory, GSI = global severity index, HRQoL = Health-related quality of life, MCS = mental component summary, PCS = physical component summary, SF-36 = Short Form-36 Health Survey, THA = total hip arthroplasty, TKA = total knee arthroplasty, WOMAC=Western Ontario and Mac Master Universities Osteoarthritis Index, norm: healthy reference group. t0: 1 day pre surgery, t1: 6 weeks post surgery, t2: 12 weeks post surgery, THA vs. norm:  *p* ≤ .008,  *p* ≤ .001. TKA vs. norm:  *p* ≤ .008,  *p* ≤ .001

Changes in all WOMAC scales for THA and TKA patients are presented separately in Fig. 1(a) and (b). There was a significant main effect for the factor time, showing an improvement of the total score and all WOMAC subscales from t0 to t2 with large effect sizes (pain: F(1.72) = 172.42; *p* = <.001; η^2^ = .631; stiffness: F(1.59) = 63.75; *p* < .001; η^2^ = .387; function: F(1.57) = 131.79; *p* < .001; η^2^ = .566; total: F(1.57) = 156.90; *p* < .001; η^2^ = .608). Also significant main effects for the factor group (THA vs. TKA) were detected for the subscales pain (F(1) = 7.12; *p* = .009; η^2^ = .066) and stiffness (F(1) = 5.02; *p* = .027; η^2^ = .047), with TKA patients having worse values at all time points on both scales. Furthermore, there were significant interactions between time and group for the subscales pain (F(1.72) = 5.61; *p* = .006; η^2^ = .053) and stiffness (F(1.59) = 4.94; *p* = .014; η^2^ = .047) as well as for the total score (F(1.57) = 4.23; *p* = .024; η^2^ = .040). On the subscale pain, THA patients improved significantly from t0 to t1 (*p* = <.001) and from t1 to t2 (*p* = .030), whereas scores of TKA patients decreased significantly from t0 to t1 (*p* = <.001) but remained stable from t1 to t2 (*p* = .741). Regarding stiffness, THA as well as TKA patients showed significant improvements from t0 to t1 (THA: *p* < .001; TKA: *p* = .001) but did not change from t1 to t2 (THA: *p* = 1.00; TKA: *p* = .054). On the total score, again both groups improved significantly from t0 to t1 (both: *p* < .001), but from t1 to t2, scores further decreased for THA patients (*p* = .021) but not for TKA patients (*p* = .069). For both groups, the improvement in all three scales from t0 to t2 was highly significant (all: *p* < .001).

### Changes in general HRQoL

A significant main effect for the factor time was found for PCS (F(1.83) = 16.37; *p* < .001; η^2^ = .142) as well as for MCS scores (F(1.80) = 4.39; *p* = .017; η^2^ = .042) (Fig. 1(c)), demonstrating increasing values over time. For neither of the scales, the analyses revealed a significant main effect for the factor group. The interaction between time and group was significant only for MCS (F(1.80) = 5.67; *p* = .006; η^2^ = .054). In THA patients, MCS did not change from t0 to t1 (*p* = 1.00), but increased significantly from t1 to t2 (*p* = .039), whereas in TKA patients, the trend was contrary (t0 to t1: *p* = .001; t1 to t2: *p* = .264). Changes resulted in an overall improvement of MCS from t0 to t2 in TKA patients (*p* = .036) but not in the THA group (*p* = .502).

The comparisons of THA and TKA patients to a healthy reference group [20] can be seen in Fig. 1(c).

### Changes in psychological distress

The anxiety (F(1.09) = 10.31; *p* < .001; η^2^ = .093) and the depression subscale (F(1.92) = 3.97; *p* = .022; η^2^ = .039) as well as the GSI (F(1.91) = 9.963; *p* < .001; η^2^ = .089) demonstrated significant main effects for the factor time, indicating improvements from t0 to t2 on all three scales (Fig. 1(d)). Neither significant main effects for the factor group nor significant interactions between time and group could be detected for any of the scales.

Figure 1(d) presents the comparisons to a healthy reference sample [22].

### Changes in sense of coherence

The analyses showed a significant main effect for the factor time (F(1.93) = 6.271; *p* = .003; η^2^ = .064) as SOC-13 scores increased from t0 to t2. No significant main effect of the factor group and no interaction between time and group have been found.

At t0, scores of THA (*t* = 1.68; *p* = .101) as well as TKA patients (*t* = 1.014; *p* = .315) were comparable to a representative reference sample [25], whereas at t1 and t2, both groups showed significantly higher scores (t1: THA (*t* = 3.43; *p* = .001), TKA (*t* = 2.90; *p* = .005); t2: THA (*t* = 3.56; *p* = .001), TKA (*t* = 3.76; *p* < .001)).

### Changes in social support

A significant main effect for the factor time was found, indicating an increase of social support over time (F(1.48) = 4.07; *p* = .030; η^2^ = .038). Neither a significant main effect for the factor group nor an interaction between time and group could be detected.

THA as well as TKA patients exhibited significantly higher scores than a representative reference sample [24] at all three time points (t0: THA (*t* = 4.30; *p* < .001), TKA (*t* = 3.57; *p* = .001); t1: THA (*t* = 6.97; *p* < .001), TKA (*t* = 6.01; *p* < .001); t2: THA (*t* = 7.25; *p* < .001), TKA (*t* = 5.46; *p* < .001)).

### Identification of predictors

For THA and TKA patients, separate regression analyses have been calculated for t2-scores of WOMAC total as well as all WOMAC subscales. As possible predictors, baseline BSI anxiety, BSI depression, SOC-13, F-SozU and the baseline score of each model’s dependent variable have been included. Results of the regression analyses are shown in Table 2.

**Table 2:** Regression analyses for THA and TKA patients

	Dependent variable	R^2^ corrected	Significant predictors	B	SE B	β	t	p
THA	t2 WOMAC pain	.171	t0 BSI anxiety	1.52	0.49	.44	3.11	.003
	t2 WOMAC stiffness	.214	t0 WOMAC stiffness	0.32	0.11	.38	2.78	.008
			t0 SOC-13	−0.82	0.35	−.32	−2.32	.026
	t2 WOMAC function	.316	t0 BSI anxiety	1.89	0.51	.48	3.69	.001
			t0 F-SozU	−0.72	0.35	−.27	−2.08	.044
	t2 WOMAC total	.305	t0 BSI anxiety	14.06	3.91	.47	3.60	.001
			t0 F-SozU	−5.38	2.64	−.27	−2.04	.048
TKA	t2 WOMAC pain	.137	t0 WOMAC pain	0.36	0.12	.39	3.13	.003
	t2 WOMAC stiffness	.096	t0 BSI anxiety	1.17	0.45	.34	2.61	.012
	t2 WOMAC function	.183	t0 WOMAC function	0.45	0.12	.45	3.65	.001
	t2 WOMAC total	.195	t0 WOMAC total	0.46	0.12	.46	3.79	<.001

Legend: *THA* total hip arthroplasty, *TKA* total knee arthroplasty, *WOMAC* Western Ontario and Mac Master Universities Osteoarthritis Index, *BSI* Brief Symptom Inventory, *SOC*-13 Sense of coherence scale-13, *F-SozU* questionnaire of social support

Independent variables in the regression model: t0 BSI anxiety, t0 BSI depression, t0 SOC-13, t0 F-SozU, t0-score of the model’s dependent variable

Regarding THA patients, the only significant predictor for WOMAC pain was anxiety, showing a positive relationship. WOMAC stiffness was predicted by the baseline score in a positive direction as well as by SOC-13 in a negative direction. Significant predictors for WOMAC function were anxiety and social support. Higher functional limitations were predicted by higher anxiety and less social support. The same two predictors, in the same directions, revealed significant for WOMAC total.

In the group of TKA patients, for the WOMAC subscales pain and function as well as for the total score the respective baseline scores revealed as only significant predictors. Each outcome variable was positively correlated to its baseline score. WOMAC stiffness was predicted by anxiety, showing a positive relationship. For all outcome parameters in both groups, the amount of explained variance ranged between 9.6 and 31.6%.

## Discussion

This study compared in a prospective design outcomes of patients after THA with those after TKA and investigated which psychosocial parameters had an influence on outcomes in each group. To our knowledge, it is the first study to define predictors for each procedure separately and to contrast them.

As expected, THA as well as TKA patients showed high improvements in disease-specific variables and in general HRQoL after surgery, consistent with the existing literature. Because most variables did not differ between patient groups before surgery, comparability of changes was warranted. Overall, in our study the improvements seen in the WOMAC scores were even stronger for the THA than for the TKA group, which replicates the results of other studies regarding joint-specific instruments [3, 6, 14, 15]. Beyond that, in both patient groups we saw a reduction of psychological distress as measured by the three BSI scales and an increase in psychosocial resources, namely sense of coherence and social support. Whereas the decrease in anxiety and depression is in line with the literature [7, 11, 16, 26, 27] a change in SOC or social support has rarely been investigated [7]. The strong relationship of SOC to perceived health has widely been proven [8] and social support has shown to be an essential protective factor against psychological strain for example after traumatic experiences [28]. So it was our goal to examine the role of these two important constructs in the context of total joint arthroplasty. We know only one study which investigated SOC as predictor after surgery and this was restricted to THA, not including TKA patients [11]. Social support has been studied in different investigations but with conflicting results [4, 7, 29, 30].

Our main finding, which adds to the existing literature, was the difference between THA and TKA patients regarding the relationship of WOMAC with psychosocial variables. With one exception (stiffness baseline score predicted stiffness at 12 weeks), in THA, the regression analyses with all WOMAC scales as dependent variables solely revealed psychosocial parameters as significant predictors and WOMAC baseline scores seemed to be negligible. In contrast, in TKA patients, almost all scales were predicted by the baseline scores of the corresponding scales and with one exception (anxiety as predictor for stiffness) psychosocial variables did not play a role in predicting disease-specific outcome. This suggests that the relationship between physical and psychological aspects is much greater in THA patients. Maybe, the different results for the stiffness scale are due to its lower reliability compared to the pain and function scales because it only consists of two items.

The literature regarding psychosocial variables as predictors for physical outcome after THA or TKA is highly inconsistent. For this reason, we aimed to provide more evidence concerning this topic. In TKA patients, the results of other studies are heterogeneous. Desmeules et al. [31] found psychological distress (anxiety and depression) to influence SF-36 physical functioning but not WOMAC pain and function. Social support did not predict either of the scales. A review by Lewis et al. [32] identified depression, anxiety and social support as predictors for pain. In a study by Lopez-Olivo et al. [7] depression and one form of social support influenced WOMAC function but not WOMAC pain. Anxiety did not reveal as predictor for function or pain in this study. Depression predicted neither WOMAC function nor WOMAC pain in a study by Sullivan et al. [33]. Beyond anxiety, depression and social support, which were also investigated in our study, different coping styles, locus of control [7], (pain) catastrophizing [32, 33] and mental health [32] revealed as predictors for some physical variables but not for others in different investigations. However, despite all these conflicting findings, the baseline score of a particular physical variable nearly always predicted the outcome score after TKA [16, 31–33], which confirms the result of our study. With respect to THA, we found only few studies investigating the influence of psychosocial variables on physical outcome after surgery. Quintana et al. [30] found baseline scores and mental health but not social support to influence WOMAC and SF-36 scores. Sense of coherence, anxiety as a trait and neuroticism but not anxiety as a state, depression or extraversion predicted SF-36 PCS and MCS in a study by Badura-Brzoza et al. [11]. Only partially in accordance with that, we identified anxiety, social support and sense of coherence as predictors for the different WOMAC scales in our study. But as all these investigations show significant methodical differences they are difficult to compare.

As mentioned above, in TKA, the influence of physical baseline scores on the corresponding outcome is the most consistent finding [31]. From our results we conclude, that they even have the strongest effect on physical outcome after surgery. In contrast, the impact of psychosocial variables seems to be much weaker in this group. Even if we had detected additional psychosocial predictors with a larger sample size, physical baseline scores still would seem to play the most important role. It is the strength of our study that we investigated TKA as well as THA patients, so we found out that in THA, the results are contrary. In this group we saw a stronger influence of presurgery psychosocial parameters than of physical baseline scores on the physical outcome.

These findings could possibly be explained by the differences in recovery after both procedures. Several studies pointed out that in both groups the greatest improvements occur in the first 3 to 6 months after surgery followed by slighter improvements thereafter [6, 13, 16, 34]. But even if THA and TKA patients lastly reached a similar level of specific or general HRQoL, THA patients showed faster recovery than TKA patients [3, 6, 34]. Dailiana et al. [16] reported the same trend for WOMAC pain and stiffness but not for WOMAC function and total score, in which the TKA group showed higher improvements (on both scales, TKA patients already exhibited better baseline scores). Furthermore, patients who underwent both, THA and TKA, reported more postoperative pain, lower improvements in range of movement and in quality of life, longer time before they felt physically better than they had before surgery and more required physiotherapy after TKA than after THA. THA was only rated inferior to TKA regarding the longer acute hospital length of stay [15]. So, patients after THA were more overall satisfied than TKA patients [3, 15, 34, 35] and more willing to undergo another total joint arthroplasty [15, 35]. It can therefore be concluded that TKA is a more difficult procedure than THA with a longer and more complicated recovery process. This could be the reason why a better physical status presurgery constituted a predictive factor for a better physical outcome after surgery in this group. Possibly, psychosocial factors also had an influence in TKA, but this was overlapped by the high impact of WOMAC baseline scores. Instead, in THA, results seem to be more independent from baseline physical conditions, at least in the early recovery process, so psychosocial parameters´ effects are more evident. Indeed, TKA patients showed psychological distress as indicated by significantly higher BSI GSI scores compared to the THA group, both before and after surgery. The worse prognosis and concerns of possible complications might have been the reason for higher distress in this group already prior to operation. Maybe patients informed themselves about the planned intervention and TKA patients found more discouraging information than THA patients. After arthroplasty, the higher distress presumably was related to the inferior physical condition.

As the follow-up period in our study took no more than 3 months, patients experienced a critical phase in recovery. If we had investigated our sample 6 or 12 months later, it could have been possible that the observed differences between THA and TKA patients regarding predictors for physical outcome scores no longer would have been found. Suggestive of this possibility are the results of two studies which investigated THA and TKA patients together. Serra-Sutton et al. [4] found that THA as well as TKA patients with low baseline scores in the SF-36 subscale mental health presented worse values in all SF-36 and all WOMAC scales 1 year after surgery. In the study by Dailiana et al. [16], in both patient groups predictive variables for changes in WOMAC scales 12 months after surgery were the corresponding baseline scores (with the exception of stiffness in TKA), whereas none of the investigated sociodemographic variables had an influence.

Overall, in our study, from all psychosocial variables tested, anxiety most often revealed as a significant predictor and showed elevated preoperative levels at least in the TKA group. So in preparing for total joint arthroplasty, distressed patients should be offered interventions for minimizing anxiety (for example psychosomatic contacts) to reveal a better outcome after surgery. Also, to a lesser extent, social support and sense of coherence influenced outcome in the THA group. In our study, the patients showed average or even above average scores in both parameters, but other patients could have a need to improve sense of coherence before surgery or could benefit from contacts to social services.

A limitation of our study is the relatively short follow-up period of 12 weeks. A longer follow-up period would have provided the opportunity to analyze if the identified differences between THA and TKA patients will maintain over time or maybe decrease. However, as mentioned above, the most significant changes occur shortly after surgery, so this was the phase we were primarily interested in. Furthermore, we did not investigate possible confounders like comorbidities or BMI. So other studies with greater sample sizes and controlling for different confounders are needed to further test our hypothesis.

## Conclusions

To our knowledge, this is the first study to contrast predictors of physical outcome after surgery in THA vs. TKA patients. Our results suggest that although disease-specific dimensions and general HRQoL as well as psychological distress and psychosocial resources show great improvements after a total joint arthroplasty, the improvements in physical outcome are predicted by different variables in THA and TKA in the early recovery process. Whereas in the first 3 months after surgery physical outcome seems to be stronger influenced by physical baseline scores in TKA patients, in THA patients it seems to be stronger influenced by psychosocial aspects. However, TKA patients also reported mental distress before and after surgery, so patients in both groups, who exhibit a respective need, should be offered interventions to reduce psychological distress.

## Abbreviations

BSI: Brief Symptom Inventory
F-SozU: Questionnaire for social support
GSI: Global severity index
HRQoL: Health-related quality of life
M: Mean
MCS: Mental component summary
PCS: Physical component summary
SD: Standard deviation
SF-36: Short Form-36 Health Survey
SOC: Sense of coherence
SOC-13: Sense of coherence scale-13
THA: Total hip arthroplasty
TKA: Total knee arthroplasty
WOMAC: Western Ontario and Mac Master Universities Osteoarthritis Index
